# Supplementary material for: Essential Oil of Algerian Eryngium campestre: Chemical Variability and Evaluation of Biological Activities
Source: Molecules. 2019 Jul 15;24(14):2575. doi: 10.3390/molecules24142575 (PMC6680772; doi:10.3390/molecules24142575)
Supplement: Supplementary file 1 [file molecules-24-02575-s001.pdf]

Table (supplementary material): Chemical Composition of *Eryngium campestre* Collective Essential Oil (EC-CO) isolated from the Aerial Parts and Essential-Oil Samples S1 –S37 (S min, S max) from western of Algeria.

No.[a]	Components	*l*RIa [b]	RIa [c]	RIp [d]	EC-CO[e]	Smin	Smax	Lalla setti				Bouhanak	Mafrouch		Zenata	Remchi
								S1	S2	S3	S4	S5	S6	S7	S8	S9
1	β-Pinene	970	972	1110	0.1	0.1	0.4	0.3	0.2	0.1	0.4	tr	tr	tr	tr	tr
2	Myrcene	979	982	1153	2.7	0.1	8.4	8.4	3.0	1.3	4.7	3.5	3.4	2.4	4.9	4.6
3	p-Cymene	1011	1013	1258	tr	0.1	4.4	tr	0.2	0.2	tr	0.3	tr	tr	tr	0.1
4	Limonene	1020	1022	1199	0.1	0.1	2.0	tr	tr	tr	tr	tr	0.1	tr	tr	tr
5	(Z)-β-Ocimene	1024	1026	1230	0.1	0.1	0.2	tr	tr	tr	tr	tr	tr	tr	tr	tr
6	δ-Terpinene	1047	1049	1243	tr	0.1	0.3	0.1	tr	0.2	tr	tr	tr	tr	tr	tr
7	Nonan-2-one	1070	1076	1388	tr	0.1	0.2	0.2	tr	0.1	tr	tr	tr	tr	tr	0.1
8	Terpinolene	1078	1079	1280	tr	0.1	0.4	0.2	0.3	tr	0.1	tr	0.1	0.1	0.1	tr
9	Nonanal	1083	1082	1394	0.1	0.1	0.5	0.1	0.2	0.1	0.1	0.1	tr	tr	0.1	tr
10	Decanal	1185	1184	1498	0.3	0.1	0.8	0.2	0.6	0.3	0.2	0.1	0.2	0.4	0.1	tr
11	(E)-2-Decenal	1248	1247	1652	0.4	0.1	0.7	0.3	0.2	0.1	tr	tr	0.4	0.3	0.1	0.2
12	(E)-2-Undecanal	1343	1348	1726	tr	0.1	0.4	0.1	0.1	tr	0.2	tr	0.1	tr	tr	tr
13	α-Copaene	1379	1375	1438	0.9	0.1	1.4	1.1	0.3	0.3	1.4	1.0	1.4	0.6	0.3	tr
14	β-Bourbonene	1385	1383	1515	0.1	0.1	2.9	0.1	0.6	0.1	0.2	tr	0.2	0.1	0.1	1.2
15	β-Elemene	1388	1387	1589	3.0	0.1	8.8	1.5	0.1	4.2	1.9	tr	1.5	1.2	2.2	0.1
16	β-Ylangene	1420	1417	1562	0.6	0.1	0.9	0.8	0.4	0.7	0.9	tr	1.1	0.9	0.6	1.6
17	(E)-β-Caryophyllene	1424	1426	1591	tr	0.1	1.6	0.3	0.1	1.5	0.4	tr	1.2	0.7	0.2	1.2
18	δ-Elemene	1431	1432	1581	1.2	0.1	7.4	0.7	0.1	0.1	tr	tr	0.1	tr	7.4	3.8
19	trans-α-Bergamotene	1432	1433	1575	1.2	0.1	0.7	0.2	tr	tr	tr	tr	tr	tr	tr	0.7
20	(E)-β-Farnesene	1448	1449	1661	0.4	0.1	8.2	8.2	2.3	0.2	2.0	8.1	0.5	0.5	2.4	0.4
21	Alloaromadendrene	1451	1454	1631	0.2	0.1	1.7	0.2	0.2	0.1	0.1	tr	tr	1.3	tr	tr
22	α-Humulene	1456	1457	1665	0.3	0.1	2.1	0.1	0.3	0.1	tr	tr	1.2	tr	0.1	2.1

23	4,5-di-epi-Aristolochene	1467	1465	1665	0.3	0.1	1.9	tr	1.9	tr	tr	tr	tr	0.1	0.2	0.2
24	δ-Muurolene	1467	1469	1683	0.9	0.1	1.4	tr	tr	tr	0.1	0.3	tr	tr	tr	tr
25	α-Curcumene	1470	1471	1742	1.3	0.1	3.8	0.5	tr	1.6	tr	0.2	0.3	tr	tr	3.8
26	**Germacrene D**	**1476**	**1480**	**1704**	**15.2**	**0.4**	**53.4**	**30.4**	**26.4**	**29.7**	**24.2**	**39.2**	**39.0**	**36.2**	**14.6**	**17.1**
27	β-Selinene	1483	1484	1712	0.9	0.1	3.1	0.4	0.5	0.4	0.6	0.1	0.4	0.4	0.3	3.1
28	α-Muurolene	1496	1503	1720	0.8	0.2	4.1	1.9	0.5	0.2	1.2	0.7	4.1	3.0	0.9	0.4
29	β-Bisabolene	1500	1500	1720	0.2	0.1	5.3	1.4	0.2	0.5	0.5	0.1	0.4	0.1	0.5	1.7
30	Sesquicineole	1505	1506	1737	0.5	0.1	4.1	0.2	0.9	0.1	0.2	tr	0.3	0.4	0.4	4.1
31	α-cadinene	1516	1513	1752	0.3	0.1	2.5	0.2	0.3	tr	0.1	tr	tr	0.2	tr	tr
32	β-curcumene	1509	1510	1733	0.2	0.1	7.4	0.3	0.8	0.6	0.2	tr	0.3	tr	1.3	7.4
33	δ-cadinene	1516	1514	1752	1.5	0.1	6.2	1.5	0.7	tr	1.6	0.1	6.2	1.5	0.4	tr
34	(E)-α-bisabolene	1532	1531	1753	0.5	0.1	3.3	tr	0.1	0.4	0.1	0.9	0.2	0.2	2.3	3.3
35	β-Elemol	1535	1534	2072	0.8	0.1	3.4	0.1	1.8	2.6	0.4	1.8	0.2	0.2	3.4	1.5
36	7-epi-trans-Sesquisabinene hydrate	1543	1547	1991	1.0	0.1	2.6	tr	0.5	tr	0.7	1.8	tr	0.4	tr	tr
37	Salvial-4(14)-ene-1,5-epoxide	1545	1548	1941	2.1	0.1	3.3	0.2	1.0	0.9	1.2	0.6	0.2	0.2	1.0	0.3
38	Germacrene B	1553	1551	1827	3.1	0.2	21.5	0.4	6.4	4.3	5.3	0.9	2.6	1.3	21.5	14.1
39	**Spathulenol**	**1563**	**1562**	**2103**	**4.8**	**0.1**	**7.6**	1.3	**1.5**	**1.3**	**0.7**	**0.1**	**2.9**	**3.5**	**1.5**	**0.9**
40	Caryophyllene oxide	1576	1570	1980	0.2	0.1	3.9	2.6	1.9	2.2	1.3	0.1	0.8	0.9	0.4	0.5
41	Salvial-4(14)-en-1-one	1583	1577	2005	1.8	0.1	3.6	1.0	1.7	0.4	0.5	0.7	0.2	0.3	0.2	0.1
42	Ledol	1600	1602	2030	1.6	0.1	5.1	0.5	0.6	0.6	0.1	0.4	0.4	0.6	0.2	0.2
43	1,10-di-epi-Cubenol	1610	1611	2054	1.4	0.1	2.3	0.1	1.0	2.2	1.4	tr	0.4	0.5	0.3	0.8
44	Caryophylla-4(14),8(15)-dien-5-α-ol	1626	1624	2285	0.3	0.3	4.7	0.5	1.4	0.3	2.2	0.6	0.4	0.6	tr	0.7
45	α-Cadinol	1632	1638	2169	2.3	0.3	5.5	1.0	2.1	2.1	0.9	0.3	0.4	3.1	1.1	2.5
46	**α-Cadinol**	**1645**	**1645**	**2231**	**5.5**	**0.2**	**7.6**	1.2	**1.1**	**2.1**	**1.7**	**tr**	**2.8**	**3.7**	**0.3**	**0.2**
47	Eudesma-4(15)-7-dien-1-β-ol	1663	1672	2199	3.0	0.1	7.6	0.3	1.2	2.5	1.7	0.3	2.1	1.9	0.7	0.2
48	α-Bisabolol	1681	1667	2333	0.6	0.1	5.2	1.0	0.6	0.5	0.4	0.1	0.6	0.5	0.1	0.2
49	14-Hydroxy-α-muurolene	1755	1759	2599	0.5	0.1	4.4	0.3	0.6	2.1	0.2	tr	0.2	0.4	1.3	0.3
50	14-Hydroxy-δ-cadinene	1788	1784	2607	1.0	0.2	1.4	0.4	tr	0.4	0.5	tr	0.3	0.6	0.2	0.2
51	Hexadecanoic acid	1942	1941	2930	1.2	0.1	0.8	0.1	0.4	0.2	0.1	tr	tr	0.2	0.1	0.8
52	**Campestrolide**	**2142**	**2143**	**2970**	**10.3**	**1.6**	**35.3**	**8.1**	**8.9**	**5.3**	**10.3**	**11.7**	**5.9**	**12.1**	**11.5**	**2.7**

Identification	75.8	70.1	86.3	79.0	74.2	73.2	71.0	74.1	83.1	81.6	83.3	83.4
Yields		0.1	0.2	0.2	0.2	0.2	0.2	0.2	0.1	0.2	0.2	0.2
Hydrocarbon compounds	36.1	11.3	76.1	59.7	48.6	49.6	46.6	57.2	64.8	51.4	64.1	72.6
Oxygenated compounds	39.7	9.1	64.9	19.5	25.6	23.7	24.4	16.9	18.3	30.2	19.2	10.9
Sesquiterpene coumpounds	60.5	34.5	79.3	60.9	60.1	65.3	54.9	58.4	72.9	66.1	66.4	74.9
Oxygenated sesquiterpenes	27.4	6.2	36.6	10.7	17.9	20.3	14.1	6.8	12.2	17.8	11.1	12.7
Hydrocarbon sesquiterpenes	33.1	8.2	73.1	50.2	42.2	45.0	40.8	51.6	60.7	48.3	55.3	62.2
Monoterpene coumpounds	3.0	0.1	9.0	9.0	3.7	1.8	5.2	3.8	3.6	2.5	5.0	4.7
Non terpenic coumpounds	12.3	1.7	36.2	9.1	10.4	6.1	10.9	11.9	6.6	13.0	11.9	3.8

Maghniya	Chlaida	Tirni		Bni aad	Ain lekbira	Mdig	Sebdou			Abed	Bni behdel		Sidi bounoir		Sebdou/Sid djillali	
S10	S11	S12	S13	S14	S15	S16	S17	S18	S19	S20	S21	S22	S23	S24	S25	S26
tr	tr	0.3	0.1	tr	tr	0.1	tr	0.1	0.1	tr	tr	tr	tr	tr	tr	tr
1.9	4.9	5.2	1.8	0.1	4.6	2.7	2.2	4.2	5.3	1.6	2.4	2.7	1.6	1.2	0.6	1.7
tr	tr	tr	tr	tr	tr	tr	0.1	0.1	0.4	tr	0.1	0.1	tr	tr	2.2	tr
tr	1.2	0.1	0.1	tr	tr	tr	0.1	0.1	0.1	tr	0.1	0.1	tr	tr	1.1	tr
tr	0.1	tr	tr	tr	tr	0.1	0.1	tr	0.2	tr	tr	0.1	tr	tr	tr	tr
tr	0.2	tr	tr	tr	tr	tr	tr	tr	tr	tr	0.1	0.1	tr	tr	0.2	tr
tr	tr	tr	tr	tr	tr	tr	tr	0.1	0.1	tr	0.1	tr	tr	tr	tr	tr
tr	tr	tr	tr	tr	tr	tr	0.2	0.1	tr	0.1	0.1	0.2	tr	0.1	tr	tr
tr	0.3	tr	tr	tr	tr	tr	0.2	0.2	0.5	tr	0.2	0.2	tr	tr	0.3	tr
0.2	0.1	0.1	tr	tr	0.2	0.1	0.6	0.5	0.7	0.2	0.7	0.6	tr	0.2	0.1	tr
0.2	0.2	0.1	tr	0.1	tr	0.3	tr	0.1	0.2	0.5	0.2	0.4	0.3	0.4	0.2	tr
0.1	tr	tr	tr	tr	tr	tr	tr	0.1	0.1	0.1	0.1	0.2	tr	0.1	tr	tr
0.8	0.5	1.0	0.7	tr	tr	tr	0.5	0.5	0.3	1.3	0.5	0.3	0.1	0.8	0.7	tr
tr	0.2	0.2	0.2	0.8	1.0	2.9	0.1	0.3	0.2	0.7	0.3	0.2	tr	0.2	0.3	1.1
1.0	0.7	1.1	1.0	0.2	0.5	tr	1.2	2.4	1.7	0.7	1.0	0.6	1.1	0.9	0.8	tr
1.1	0.6	1.5	1.0	1.2	0.8	50.9	0.4	0.4	0.1	0.8	0.5	0.3	1.0	0.9	0.6	0.6

0.6	tr	0.4	0.6	1.6	0.7	0.4	0.1	1.5	0.1	0.8	1.4	0.8	0.6	0.4	0.2	1.2
tr	0.2	tr	tr	0.3	0.6	0.2	2.7	0.1	0.2	0.1	tr	tr	tr	tr	tr	0.5
tr	0.1	0.1	tr	tr	0.2	0.1	tr	tr	tr	tr	tr	tr	tr	tr	tr	0.7
0.6	0.6	0.7	0.8	tr	3.0	0.6	0.4	0.3	0.1	0.3	3.0	2.6	1.8	1.3	0.1	2.7
1.7	tr	tr	tr	tr	tr	tr	tr	0.3	tr	1.2	0.4	0.1	1.0	1.4	tr	1.6
0.1	0.3	tr	tr	0.5	tr	0.1	tr	tr	tr	tr	tr	tr	0.1	tr	0.2	tr
tr	0.7	tr	tr	tr	tr	0.2	0.3	0.5	0.4	tr	tr	0.1	tr	0.1	0.8	0.6
tr	tr	tr	tr	0.5	0.5	tr	1.2	tr	tr	0.2	tr	tr	tr	tr	0.9	0.2
tr	tr	tr	tr	tr	tr	0.5	0.5	1.7	1.4	0.8	1.7	0.1	tr	0.8	tr	0.9
**42.1**	**27.0**	**52.4**	**37.0**	**53.4**	**39.2**	**11.5**	**6.6**	**7.5**	**5.2**	**11.1**	**4.7**	**5.2**	**22.8**	**13.9**	**18**	**22.6**
0.4	1.0	0.5	0.5	0.5	0.3	1.2	0.6	1.7	0.4	0.3	0.2	0.9	0.5	0.4	1.5	0.3
3.2	1.4	3.0	1.6	0.3	0.4	2.5	0.3	0.2	0.2	0.3	0.2	0.2	3.1	0.6	1.0	0.4
0.1	0.3	0.5	0.2	0.7	1.4	1.1	0.4	0.2	0.2	0.1	5.3	3.7	0.2	0.4	0.3	0.3
0.4	2.6	0.3	0.4	0.5	0.5	0.1	0.4	0.5	0.5	0.6	0.4	tr	0.6	0.5	2.9	tr
0.2	0.4	tr	tr	tr	tr	tr	0.1	tr	0.1	0.1	0.7	tr	0.2	0.1	0.3	0.7
tr	tr	tr	tr	tr	tr	tr	0.6	0.1	0.4	0.2	0.5	tr	tr	0.2	tr	tr
1.5	1.0	1.7	1.5	1.9	1.3	0.5	0.6	0.7	0.3	1.9	0.7	0.5	1.9	1.2	0.8	0.4
0.1	0.4	0.2	0.3	0.3	tr	0.1	0.3	0.4	1.4	0.5	tr	tr	0.3	0.3	tr	tr
tr	tr	tr	0.1	tr	tr	tr	2.2	1.4	tr	0.2	1.0	1.0	0.2	0.1	tr	0.3
0.5	tr	0.3	0.4	0.6	0.6	tr	tr	0.8	0.2	0.8	tr	tr	0.7	0.1	1.1	tr
0.6	0.4	0.1	1.1	2.3	3.3	0.1	0.7	0.4	0.8	1.8	1.1	1.0	0.6	1.0	1.1	1.0
0.2	7.3	0.2	0.3	1.5	0.7	0.3	3.7	3.0	0.5	5.6	3.5	1.9	0.3	0.6	0.4	1.1
**3.5**	**2.1**	**1.5**	**2.4**	**0.7**	**1.1**	**1.0**	**5.9**	**2.9**	**4.9**	**6.6**	**7.6**	**6.6**	**3.8**	**6.8**	**2.8**	**3.2**
0.8	0.8	0.5	0.8	0.3	1.1	0.1	1.5	1.3	2.3	1.9	2.3	1.3	1.4	2.1	0.5	1.4
0.1	0.7	0.2	0.3	0.3	3.6	0.3	0.6	1.3	1.2	0.6	0.5	0.5	0.6	0.9	1.1	2.0
0.3	tr	0.7	0.3	0.4	2.2	0.1	0.8	0.5	2.5	2.6	1.7	0.3	1.2	1.6	tr	5.1
0.8	**tr**	0.7	0.3	1.0	1.3	0.1	1.0	1.1	1.3	0.8	0.5	0.4	0.4	1.0	**1.0**	0.6
tr	1.6	1.4	0.5	0.3	2.0	0.7	tr	0.6	0.8	4.1	0.3	tr	3.4	3.4	0.6	tr
3.1	2.7	1.3	2.8	0.5	tr	2.5	4.6	4.2	5.5	1.0	3.5	3.0	1.9	1.2	2.2	3.4
**0.8**	**1.1**	**2.0**	**3.8**	**1.6**	**1.0**	**0.9**	**7.2**	**5.1**	**5.5**	**6.4**	**1.4**	**3.1**	**6.1**	**5.1**	**4.2**	**5.1**

1.5	4.2	1.2	2.3	2.4	4.2	0.1	5.4	3.9	3.8	3.1	5.1	5.8	2.4	2.9	7.6	6.0
0.5	2.8	0.4	0.4	0.1	tr	0.2	0.6	0.5	0.2	0.7	0.7	0.6	0.5	0.7	4.6	0.5
0.5	0.1	0.3	0.5	0.3	0.3	tr	4.4	2.1	1.5	1.0	1.4	1.2	0.5	0.1	1.2	0.6
0.7	0.2	0.6	0.9	tr	0.3	tr	0.3	tr	0.2	1.1	tr	tr	0.6	1.0	0.9	1.4
0.3	0.2	0.3	0.3	tr	tr	tr	0.1	0.1	tr	0.6	0.2	tr	tr	0.2	0.5	0.3
**8.6**	**10.2**	**5.2**	**12.9**	**1.6**	**5.5**	**2.6**	**15.7**	**17**	**18**	**6.8**	**19.9**	**23.2**	**18.7**	**15.7**	**13.9**	**6.2**
79.1	79.4	86.3	78.2	76.8	82.4	85.2	75.5	71.1	70.1	70.2	76.3	70.2	80.5	70.9	77.6	74.7
0.1	0.1	0.2	0.2	0.2	0.2	0.1	0.2	0.1	0.1	0.2	0.1	0.1	0.2	0.1	0.1	0.1
56.0	51.7	69.4	49.7	64.3	55.7	76.1	26.6	29.6	20.9	32.1	30.0	22.8	38.7	27.5	36.0	38.9
23.1	27.7	16.9	28.5	12.5	26.7	9.1	48.9	41.5	49.2	38.1	46.3	47.4	41.8	43.4	41.6	35.8
67.8	61.9	75.0	63.0	75.0	72.1	79.3	56.2	48.4	44.4	60.3	52.1	42.3	59.9	53.0	58.5	66.5
14.1	19.3	11.5	17.3	11.3	21.5	6.2	35.6	26.6	31.2	33.3	27.5	24.8	24.9	28.5	31.8	30.6
53.7	42.6	63.5	45.7	63.7	50.6	73.1	20.6	21.8	13.2	27.0	24.6	17.5	35.0	24.5	26.7	35.9
1.9	6.4	5.6	2.0	0.1	4.6	2.9	2.7	4.6	6.1	1.7	2.8	3.3	1.6	1.3	4.2	1.7
9.4	11.0	5.7	13.2	1.7	5.7	3.0	16.6	18.1	19.6	8.2	21.4	24.6	19.0	16.6	14.9	6.5

Abed/Lekhmis						Boughadou	Sid Djillali		Laouadj	Lekhmis	Identification
S27	S28	S29	S30	S31	S32	S33	S34	S35	S36	S37	
0.1	tr	tr	0.4	tr	tr	tr	tr	0.3	tr	**tr**	RI. MS
0.8	2.6	1.8	2.9	2.7	5.0	4.1	1.8	4.3	1.4	0.4	RI. MS
tr	0.1	tr	0.1	tr	tr	tr	tr	tr	4.4	tr	RI. MS
tr	0.1	tr	0.2	tr	tr	tr	0.2	0.1	2.0	tr	RI. MS
tr	0.1	tr	0.1	tr	tr	0.1	tr	tr	tr	tr	RI. MS
tr	tr	tr	tr	tr	tr	tr	tr	tr	0.3	tr	RI. MS
0.1	tr	0.1	tr	tr	tr	tr	tr	tr	tr	tr	RI. MS
tr	0.4	0.1	0.1	tr	tr	0.2	0.1	tr	tr	tr	RI. MS
0.2	0.1	0.1	0.1	0.2	0.1	0.2	tr	0.1	0.1	tr	RI. MS

0.5	0.2	tr	0.1	0.7	0.5	0.6	0.5	0.4	0.8	tr	RI. MS
0.3	0.7	0.7	0.6	0.5	0.4	0.4	0.4	0.4	tr	0.5	RI. MS
0.2	0.1	tr	tr	tr	tr	0.1	tr	tr	tr	0.4	RI. MS
0.3	0.6	1.0	0.8	0.4	0.2	0.2	0.6	0.6	0.3	tr	RI. MS
0.2	0.3	0.6	0.3	0.2	0.1	0.2	0.2	0.5	0.3	0.6	RI. MS
0.7	0.9	0.7	0.9	0.4	0.6	1.2	6.7	8.8	0.7	tr	RI. MS
0.1	0.2	0.7	0.7	0.3	0.5	0.5	0.3	0.7	tr	0.2	RI. MS
0.1	0.3	0.8	0.3	0.2	0.2	0.1	0.1	0.3	tr	tr	RI. MS
0.1	tr	0.3	tr	tr	tr	4.0	0.1	0.2	tr	tr	RI. MS
tr	tr	0.1	tr	tr	tr	0.1	tr	tr	tr	tr	RI. MS
0.4	2.2	2.4	0.2	3.0	3.8	0.5	0.3	0.7	tr	0.4	RI. MS
tr	0.3	1.5	tr	0.2	0.1	tr	0.3	tr	tr	tr	RI. MS
tr	0.3	0.1	0.1	tr	0.1	tr	tr	0.4	tr	tr	RI. MS
0.7	tr	0.5	0.1	0.1	tr	0.3	tr	0.2	0.4	tr	RI. MS
tr	1.4	tr	tr	tr	tr	tr	tr	0.3	1.3	0.7	RI. MS
0.5	0.3	0.7	1.3	0.6	tr	1.1	1.1	tr	tr	0.6	RI. MS
**13.0**	**0.4**	**17.3**	**20.9**	**1.5**	**18.0**	**4.2**	**11.3**	**12.5**	**3.7**	**1.8**	RI. MS
0.2	0.2	0.4	0.2	0.3	0.2	0.6	0.9	0.7	tr	tr	RI. MS
0.3	0.3	0.6	0.5	0.5	1.3	0.4	0.6	3.2	0.3	0.5	RI. MS
0.2	1.2	0.5	0.3	0.1	0.2	0.4	0.4	0.7	1.1	0.2	RI. MS
0.4	0.6	0.1	0.5	0.1	0.2	0.3	0.4	0.3	0.4	tr	RI. MS
0.1	0.3	0.2	0.2	0.4	0.2	0.4	0.2	0.2	2.5	0.5	RI. MS
tr	0.3	tr	0.2	0.3	0.2	0.9	0.2	tr	tr	tr	RI. MS
0.5	0.3	1.2	0.8	0.5	0.8	0.2	0.4	1.0	0.4	tr	RI. MS
1.0	0.2	0.5	0.2	0.2	0.1	1.4	0.1	0.1	tr	1.1	RI. MS
0.9	tr	0.3	tr	0.1	tr	0.6	tr	0.1	tr	tr	RI. MS
0.7	tr	0.8	0.8	0.2	0.5	2.6	0.7	0.4	1.4	1.2	RI. MS
0.1	1.0	1.2	0.4	1.1	1.4	0.8	0.5	0.3	1.9	1.5	RI. MS
0.7	0.7	2.4	0.4	1.5	0.3	11.2	0.4	0.2	0.2	1.6	RI. MS
**4.0**	**6.3**	**5.0**	**4.7**	**4.3**	**2.7**	**2.6**	**4.4**	**5.3**	**3.2**	**0.5**	RI. MS

1.1	3.9	1.7	1.6	2.3	0.8	1.5	1.2	0.2	1.6	3.7	RI. MS
2.5	0.9	0.4	0.3	0.8	0.4	0.5	0.5	0.3	1.9	2.0	RI. MS
2.1	2.1	3.9	1.1	2.7	1.0	0.7	0.7	0.3	tr	3.6	RI. MS
2.3	0.6	0.6	0.3	0.4	0.3	0.6	0.3	1.2	**2.0**	0.3	RI. MS
tr	4.7	0.9	2.1	0.6	0.6	2.4	3.4	0.7	3.0	2.2	RI. MS
2.1	4.3	1.2	3.6	2.7	3.5	0.9	1.2	3.6	3.5	3.5	RI. MS
**3.9**	**5.7**	**3.6**	**5.2**	**3.8**	**2.3**	**3.0**	**6.7**	**7.1**	**7.4**	**7.6**	RI. MS
3.1	3.9	1.6	3.1	3.0	2.6	1.8	3.5	3.2	4.6	tr	RI. MS
0.8	0.8	5.2	0.6	0.5	0.6	0.8	0.9	0.3	1.4	4.5	RI. MS
0.3	0.6	0.5	0.4	0.7	0.4	4.2	0.6	0.3	2.8	0.8	RI. MS
tr	1.2	0.9	1.1	0.5	0.5	0.2	0.4	0.3	1.1	tr	RI. MS
tr	tr	0.4	0.4	0.1	tr	tr	0.3	tr	tr	tr	RI. MS
**24.5**	**18.4**	**10.2**	**15.4**	**31.5**	**30.6**	**13.3**	**17.3**	**15.5**	**14.1**	**35.3**	RI. MS
70.1	70.1	73.8	74.6	70.2	81.3	70.4	70.2	76.3	70.3	76.2	
0.1	0.1	0.1	0.2	0.2	0.1	0.1	0.1	0.1	0.2	0.2	
22.2	15.6	36.9	33.9	14.9	34.0	36.6	27.9	37.1	22.8	11.3	
47.9	54.5	36.9	40.7	55.3	47.3	33.8	42.3	39.2	47.5	64.9	
43.4	47.3	60.4	54.2	34.5	44.7	51.4	49.6	55.2	47.3	39.6	
24.3	36.6	27.9	25.8	23.8	17.8	23.5	25.4	23.9	36.2	31.4	
19.1	10.7	32.5	28.4	10.7	26.9	27.9	24.2	31.3	11.1	8.2	
0.9	3.3	1.9	3.8	2.7	5.0	4.4	2.1	4.7	8.0	0.4	
25.8	19.5	11.5	16.6	33.0	31.6	14.6	18.5	16.4	15.0	36.2	

a Order of elution is given on apolar column (Rtx-1). Bold types refer to main compounds.
b Retention indices of literature on the apolar column (lRIa).
c Retention indices on the apolar Rtx-1 column (RIa).
d Retention indices on the polar Rtx-Wax column (RIp).
e EC/CO: *Eryngium campestre* Collective oil. Quantification was carried out using RFs relative to tridecane as internal standard. %: Normalized Percentages are given on the apolar column except for components with identical RIa (percentages are given on the polar column), tr = trace (<0.05%).

f RI: Retention Indices; MS: Mass Spectrometry in electronic impact mode. Ref : comparison with literature data. All compounds were identified by comparing their EI-MS and retention indices with references compiled in the in-house library.
